# 852. Epidemiology, clinical characteristics and outcomes of invasive pulmonary aspergillosis among critically ill patients in medical intensive care unit: A retrospective study from the tertiary care hospital in Thailand

**DOI:** 10.1093/ofid/ofad500.897

**Published:** 2023-11-27

**Authors:** Noppharut Yutthana, Kamonwan Jutivorakool, Jakapat Vanichanan

**Affiliations:** Faculty of Medicine, Chulalongkorn University and King Chulalongkorn Memorial Hospital, Bangkok, Krung Thep, Thailand; King Chulalonkorn Memorial Hospital, Bangkok, Krung Thep, Thailand; Chulalongkorn University, Bangkok, Krung Thep, Thailand

## Abstract

**Background:**

Invasive pulmonary aspergillosis (IPA) is a serious infection which leads to poor outcomes particularly in immunocompromised patients. Recently, IPA in critically ill patients in intensive care unit (ICU) has been increasingly recognized owing to its substantial morbidity and mortality. However, clinical data of IPA in ICU is scarce especially in Thailand.

**Methods:**

A single-center, retrospective, observational study was conducted in King Chulalongkorn Memorial Hospital. We collected data on demographic, clinical characteristics, and outcomes of the patients who developed IPA during admission in the medical intensive care unit (MICU) between Jan 2016 to Dec 2021. IPA was identified by using ICD-10 and medication code for voriconazole, amphotericin B or liposomal amphotericin B. Patients met proven, probable IPA according to EORTC/MSG definition or putative IPA were included. The primary outcome was the incidence of IA in the MICU. Clinical characteristics, 12-week all-cause mortality and associated risk factor were the secondary outcomes.

**Results:**

During the study period, a total of 5,019 patients were admitted in MICU. Fifty-seven met definitions of IPA which represented incidence of 1.14%. Seven patients (12.3%) had proven IPA, while 37 (61.4%) and 13 (22.8%) patients had probable and putative IPA, respectively. Cardiovascular disease (33.3%), chronic liver disease (26.7%), and diabetes (20.0%) were common comorbidities in patients with putative IPA. Diagnosis was made at median of 5 (3-10) days after ICU admission. Organisms were identified in 36 patients (63%) which *Aspergillus fumigatus* was the most common species (36.8%). All-cause mortality at week 12 was 89.5%. SOFA score (OR 1.494, 95%CI 1.058-2.110) and vasopressor used on admission (OR 16.00, 95%CI 2.209-115.92) had trend toward increase mortality. Median survival in putative and probable IPA group were 4 and 8 days after diagnosis (p= 0.023). Patients who received treatment with voriconazole had median survival 13 days compared to 5 days in other antifungal agents (*p*= 0.006).

**Conclusion:**

IPA carried a high mortality among critically ill patients in MICU. High SOFA score and vasopressors used at the MICU admission were associated with mortality. Voriconazole therapy appeared to correlate with longer survival time.

**Disclosures:**

**All Authors**: No reported disclosures

